# Household decision-making around food in rural Tajikistan: a cross-sectional study to help extension workers in the field

**DOI:** 10.29219/fnr.v62.1330

**Published:** 2018-03-15

**Authors:** Elizabeth A. Wood, Katharine McNamara, Agata Kowalewska, Nargiza Ludgate

**Affiliations:** 1 Department of Environmental & Global Health, College of Public Health and Health Professions, University of Florida, Gainesville, FL, USA; 2 Department of Food Science & Human Nutrition, College of Agricultural and Life Sciences, University of Florida, Gainesville, FL, USA; 3 Department of Animal Sciences, College of Agriculture and Life Sciences, University of Florida, Gainesville, FL, USA

**Keywords:** decision-making, food misconceptions, gender, global health, nutrition

## Abstract

This study was conducted to research and develop recommendations for gender transformative approaches that will address misconceptions around food and nutrition, and reducing barriers around dietary diversity within rural Khatlon Province, Tajikistan. Most of the population in Tajikistan live in rural areas and spend a large part of their income on food. While stunting in children under 5 years has decreased, acute malnutrition and the number of underweight children has increased. This is a qualitative, cross-sectional study that involved secondary data analysis, key informant interviews (KIIs), and focus group discussions (FGDs) to gauge appropriate interventions for agricultural extension agents seeking to improve the nutritional outcomes of their communities. In February of 2017, data were collected from 4 KIIs and 15 FGDs that were stratified as mothers with young children, mothers-in-law, and husbands, across 12 different villages. Analysis of the KIIs and FGDs included NVivo software for coding and to uncover the most salient themes and characteristics from each. The participants of this study reported several misconceptions and taboos surrounding certain foods, especially during pregnancy, and food practices for children under the age of 5 years. Results also indicated a household hierarchy of decision-making surrounding food that included who buys, cooks, and decides what to buy. The findings of this study will be used as a springboard to launch gender-responsive and nutrition-sensitive interventions through the local agricultural extension agents.

Tajikistan is a landlocked, mostly mountainous country within Central Asia that borders Uzbekistan, Afghanistan, Kyrgyzstan, and China. The population in 2017 was 8.4 million people, with a 0.99 male to female ratio in 2016 and 73% living in rural areas (1, 2). Since the collapse of the Soviet Union, several countries within Central Asia, including Tajikistan, have experienced the breakdown of their economic system. According to the World Bank (3), inequality, specifically in rural regions of Tajikistan, has increased between 2012 and 2014, with the Gini Coefficient standing at 29 on a national level and 28.4 within rural areas. The gross domestic product (GDP) is gradually improving with a specific emphasis on diversifying the agricultural sector, which accounts for 23% of the GDP and provides approximately 75% of the labor force (4, 5). Despite these improvements, Tajikistan has struggled with having the highest malnutrition rates within Central Asia, with 10% of children under 5 years suffering from acute malnutrition, 26% from chronic malnutrition, and over 75% of the population living within rural Tajikistan still facing food insecurity (6).Tajikistan has four provinces (Sughd, Khatlon, Gorniy-Badakhshan, and the Region of Republican Subordination); however, Khatlon Province was specifically chosen for this study because of its potential for agricultural production, high undernutrition rates, and having the most people living in poverty (5).

The scarcity of jobs in rural Tajikistan drives over 800,000 people, mostly men, to migrate out of Tajikistan in search of employment; over 95% of people that migrate go to Russia specifically (7). Khatlon Province, the site of this study, experiences a higher rate of male migration (38.9%) than the national average (35.7%) (8). The remittances that are sent back account for over half of Tajikistan’s economy and are primarily controlled by women within households (3, 7). As a result of men leaving the household in search of job opportunities and higher wages, there has been a feminization of agriculture within rural villages. Some reports have revealed that gendered roles in agriculture, gendered division of labor, and gendered determinants of social status have gradually shifted as a result of this male migration, specifically in Khatlon Province, and have allowed women the opportunity to participate in decision-making within the household as well as control the household income (2, 7). However, other reports have indicated that ‘Despite labor migration having expanded women’s roles, it does not appear to have affected their status in terms of increased agency and ability to make autonomous personal choices’ (9, p. xvii). This gender imbalance has had other negative effects with regard to adolescent girls, aged 15 and 16 years, marrying at an earlier age to ensure that they have a husband (10). Therefore, while male-out migration may be empowering women, specifically mothers-in-law, within the household, it has created a triple burden of responsibilities for young women in terms of domestic, agricultural, and family obligations.

Malnutrition remains a public health problem within Tajikistan. In 2012, the Demographic Health Survey in Tajikistan indicated that 26% of children under 5 years are stunted, 10% are wasted, and 12% are underweight, with Khatlon Province having among the highest percentages in all three indicators (2). Malnutrition and undernutrition is most severe among vulnerable populations, such as women and children. According to a joint report by the World Bank and UNICEF (11), the immediate causes of undernutrition are inadequate dietary intake and disease, driven by inadequate access to food, inadequate care provided to children, and insufficient health and environmental services. This is also perpetuated by gender inequality, underrepresentation of girls within education, and government resource allocations that are unfavorable to the poor (11). Women specifically are more susceptible to anemia and deficiency of vitamin A and iodine which contribute to Tajikistan’s high maternal and child mortality (4, 5, 12).

The goal of this study was to research and develop recommendations for gender transformative approaches – addressing gender inequalities and transforming the dynamics between men and women – that will address food misconceptions, perceptions of healthy eating, and reduce barriers surrounding dietary diversity within the rural Khatlon Province of Tajikistan. The Agriculture Extension System (AES) is in a unique position to transmit these practices to the field through existing, community-based programs. AES agents, responsible for delivering information to farmers, have the potential to bridge agriculture, nutrition, and gender by integrating these topics within their ongoing technical trainings. The specific aims of this study were to identify and understand ways to engage husbands and mothers-in-law (who live within or near the same domicile) in household nutrition through extension services and to investigate practices within households around dietary habits and nutritional behavior. These groups were chosen specifically as a result of young women, often young or new mothers, being forced to leave school in order to take on the burden of feeding the household, being the caretaker of children, and managing the household chores, such as collecting water (9). The mother-in-law (which will be abbreviated as MiL since this study was from the perspective of the young, in-married women) plays and important role in food choice due to the hierarchy of the household. Young or new mothers (who will be referred to as in-married women) often live with or near her in-laws where she has limited agency and decision-making power within the household. The household dynamic in rural Tajikistan differs from urban areas in that the average household has 6.9 members compared to 4.9 members in urban households; this again is a result of households in rural areas being traditionally multigenerational with the in-laws living in the same domicile (2). In addition, households within Khatlon Province tend to have the lowest rates of household members who are over the age of 6 years and received any education (2). Therefore, by working within the unique community and household context of rural Tajikistan, efforts to improve nutrition will be more sustainable and have increased impact on the lives of people in the region.

## Methods

In collaboration with in-country partners, fieldwork for this study was completed in February 2017. Researchers from the University of Florida (UF), located in the United States, integrated students from the Masters of Public Health (MPH) program in Gainesville, Florida, United States, in addition to students from the Tajikistan Agrarian University (TAU) in Dushanbe, Tajikistan, to assist in the data collection and fieldwork phase of this study. Both UF and TAU students had public health or agriculture extension knowledge prior to the study and were trained in qualitative research methods before participating in any data collection. UF researchers had background knowledge in global public health and nutrition. A local partner, Tajikistan Agriculture and Water Activity (TAWA), assisted with logistical support, such as lodging, and provided trained, local agriculture extension agents to facilitate focus groups.

### Participants

Participants for the KIIs were selected based on their work within the Khatlon Province or their knowledge of the population as it pertains to nutrition, including a clinic director and representatives from international organizations. Participants for the FGDs were purposefully recruited from villages that extension agents work in and have established women’s groups. These women’s groups, formed by the Women Entrepreneurship for Empowerment Project (WEEP), bring women of reproductive age together to learn skills, specifically related to agriculture, and nutrition-related aspects. TAWA extension agents have been working throughout these rural villages, establishing trust and relationships with the women inhabitants. Both KIIs and FGDs were conducted to triangulate individual and group-level perception on food, nutrition, and household decision-making within Khatlon Province. However, given the experience of the key informants working in nutrition and health in the target area, more emphasis was put on lessons learned from their fieldwork. Prior to initiating the FGDs, the instrument was tested in Yovon, a village within Khatlon, among mothers with children under the age of 10 years. The instrument was revised and adjusted appropriately for cultural sensitivity.

### Study design

KIIs were conducted in English or Tajik by UF researchers, UF masters students, and TAU students. During both KIIs and FGDs, an UF researcher was present to ensure quality of data collected. The TAU students acted as translators during interviews conducted in Tajik, while also asking relevant probing questions when appropriate. Interviews were open-ended and semi-structured, and content included perceptions of household nutrition and decision-making surrounding food within the rural villages of Khatlon. Verbal informed consent was requested and documented prior to the interview. A total of four KIIs were conducted, which included interviews with the World Health Organization, UNICEF, German Corporation for International Cooperation, and a local health clinic within Khatlon Province. Key informants were initially chosen based on the in-country partners’ knowledge of international organizations working in nutrition. Key informants also provided information on best practices for data collection within Khatlon Province and what to expand on from their previous experience and research.

A total of 15 FGDs were conducted with participant groups ranging from 5 to 18 individuals; however, two focus groups were excluded because of size and participant makeup.^1^ Focus groups were stratified as young mothers with children (in-married woman), the in-married women’s MiL, and husbands of the in-married women, throughout the catchment area. These groups were chosen based on the composition of the household in Khatlon Province. It was strongly recommended by key informants to keep these groups separated for honest responses. Focus groups were asked semi-structured questions regarding decision-making surrounding food and dietary behavior. Each female FGD was facilitated by a trained agriculture extension agent with a UF and TAU student present. Adjacent to the focus group, the TAU student translated the dialogue between the facilitator and the group in real-time so that the research team could transcribe the conversation as well as ask clarifying questions (via the facilitator) to participants. All male FGDs were conducted by a male TAU student with at least one UF student present. Given the high rate of illiteracy within the study population, informed consent for focus group participants was collected verbally before the discussion began and was read in the local language (Uzbek or Tajik). Focus groups took place in 12 different villages throughout Khatlon Province, and were chosen based on differing geographic barriers and socio-economic status within USAID’s Zone of Influence (ZOI). Fifteen FGDs were conducted, with 13 being homogenous by sex and age group, and 2 being mixed ages as previously indicated (seven focus groups with in-married women, four with MiLs, two with husbands, and two with mixed MiLs and in-married women).

### Qualitative analysis

Transcripts from 4r KIIs and 13 out of 15 FGDs were used for data analysis. Researchers carried out the qualitative analysis using the constant comparative method (13) to code transcripts and find emerging themes simultaneously. Grounded theory (14) was used within analysis to contextualize the themes as they materialized in order to develop connections and relationship that were subsequently made into subthemes. Two of the researchers independently read through all of the transcripts for salient themes, subsequently forming a consensus based on frequency of themes and discussion. All transcripts were then coded independently by two researchers to ensure reliability. All of the final manifested themes and subthemes were centered on household decision-making, food beliefs, and health to better understand how to design applicable interventions with the TAWA extension agents. Themes and subthemes were collected from the data deductively and inductively in that certain themes were created *a priori* whereas others, and most subthemes, were identified upon review using QSR International’s NVivo 11 software. Once themes and subthemes were identified, they were presented to the UF research team to finalize and establish a consensus.

## Results

There were a total of 106 participants across 13 FGDs within five districts (Shaatruz, Jomi, Khuroson/Ghozimalik, Sarband, and Vakhsh) of Khatlon Province. The results of the FGDs have been analyzed and presented below according to the following major themes: Decision-making around food (Theme 1), accessibility of food (Theme 2), misconceptions around food (Theme 3), and dietary information (Theme 4). The results of the focus groups of the same participant group (in-married women, MiLs, or husbands) were highly consistent across districts with the exception of husband’s responses for Theme 3 and Theme 4. In general, greater diversity was observed in the responses between participant groups than within participant groups. Theme 3 (Misconceptions around food) and Theme 4 (Dietary information) showed the greatest variation in responses between in-married women, MiLs, and husbands (see Tables 1 and 2). Results from the KIIs were used to refine study design based on cultural practices in the region before initiating FGDs as well as to form a consensus among major themes within FGDs.

**Table 1:** Misconceptions around food for pregnant women and children under five

Misconceptio s around food	In-married women	MiLs	Husbands	Outcomes
Pregnant women	‘It depends on the state of health; when they have morning sickness they don’t eat oily meals’. ‘They don’t eat *mantou* a lot, noodles; they are told if you eat these kinds of foods or meals you will have difficulty during birth’. ‘In order not to get fat and fat on the child, we don't eat too much bread and baked goods’. ‘Doctors said that they shouldn’t eat’. ‘Doctors tell them not to eat nuts, noodles, bread, foods rich with carbohydrates and recommend to eat more fruits and juice’. ‘We know that fruits and vegetables have a lot of fertilizer and chemicals’.	‘Women shouldn’t eat noodles and foods with carbohydrates while pregnant. It makes [the] baby very big and difficult to give birth’. ‘They are told not to eat pistachios and nuts because they think the babies will be fat’. ‘They eat meat, but less eggs because they make the babies big’. ‘Pregnant women don’t eat as much oily food. They eat a lot of dairy and milk’. ‘Bakery, the pregnant women they don’t eat much baked goods like bread but they eat most legumes, meats, [and] fruit. The baby will be big and they cannot easily deliver [if they eat baked goods]. ‘Our pregnant women are afraid to get very big, and they don’t eat *osh*, noodles, *mantou.* They are told that their babies will become very big. Doctors told them’.	‘Pregnant women eat everything. Meat of horse they do not eat. Also meat from rabbits. When the baby is born, they will have holes in their lip; cleft lip or cleft palate’ ‘Pregnant women don't eat *osh’*.	In-married women, MiLs, and husbands cite restrictions on women's carbohydrate intake during pregnancy. The most common justification for this practice (given by MiLs and in-married women) is that carbohydrates make the baby large and hard to deliver (potentially macrosomia). Both MiLs and in-married women identify doctors as their source of information on avoiding carbohydrates. MiL and in-married women also discuss avoiding oily or ‘heavy’ foods during pregnancy. MiL and in-married women also note avoiding eating nuts. There is a mixed consensus on egg consumption among MiL, with some recommending egg consumption while others emphasizing meat consumption over eggs. Husbands mention avoiding horse and rabbit meat due to increased risk of cleft palate. Husbands were the only group to say that pregnant women eat everything (in-married women state that some women eat everything during pregnancy, against the advice of others)
Children under five	We don’t give them tomatoes, cucumbers, watermelons, or grapes because of diarrhea’. ‘When we make soup we make the potato and then we make it watery and make the mashed potato and give it to our children. Some women who don’t have enough breast milk, they give cow’s milk to their children, some women give formula’. ‘We don’t give fried potatoes to young children and we don’t give them *sambusa, osh, mantou*, hard meals, because it’s difficult to digest these meals’. ‘For example, one of my daughters likes *chakka* (yogurt mixture), I usually give her light meals like noodles and soup but she likes sweets’. ‘They don't give spicy foods to children under five because it is not healthy for the stomach and kidney’. ‘The water may get them sick’. ‘My children don’t eat eggs and meat; they just don’t like it’. ‘Unwashed vegetables they will get sick from greenhouses. [It] gives children diarrhea’.	‘We don’t give cucumbers to the children. We don’t give boiled eggs because it’s very hard for the stomach to digest, it makes them [have] diarrhea. Some of them after they eat cucumber, they drink water (not boiled)’. ‘For children under 2 years old, we do not give them *osh*. We do not give them spicy foods or hot meals’. ‘Babies who didn’t start talking, they shouldn’t eat eggs, because it will influence. They will start speaking very late’.	‘[Children under 5 don’t eat] spicy food. Salty food.’ ‘Eggs. It has many calories. Fish, because of the small bones.’	There are differing opinions of best child-feeding practices among in-married women, MiLs, and husbands. One consensus, however, is the avoidance of feeding children eggs. Each group cites a different reason for not feeding their children eggs. In-married women and MiLs voice concern over foods that may cause diarrhea; both groups cite cucumbers as a source of diarrhea. In-married women mention additional foods (tomato, watermelon, and grapes) and unwashed vegetables/fruits as potential sources of diarrhea. MiLs and in-married women also agree that children should not be given *osh, mantou*, eggs, or high-oil foods. In-married women strongly emphasize the importance of preparing light meals or foods with soft texture (soaked biscuits, mashed potatoes, soups) for children. In-married women also note the importance of general child health as a determinant for feeding practices (if the child is already healthy, he/she will experience little enteric disease; if he/she is naturally sickly, there will be more frequent diarrhea) and child food preference factors into what the in-married women prepares for her children. These factors were not cited by MiLs or husbands.

**Table 2:** Dietary information

Dietary Information	In-married women	MiLs	Husbands	Outcomes
Cause illness	‘I don’t eat eggs. I have an allergy and it doesn’t matter how it’s used’. ‘She has allergies so she doesn’t eat legumes because of the allergy. She doesn’t eat eggs; she doesn’t eat kidney beans, [because of] allergies on her hands and feet. She went to the doctor but nothing’. ‘All of the vegetables and foods which are grown with chemicals and fertilizer, they can affect us and they can make us sick. Some people died from botulism, after they ate the canned product last year there was one case of botulism’. ‘Some people when they eat melons they have allergies’. ‘In summer [children] when they eat grapes it makes them diarrhea. Or if they eat melons it also makes them diarrhea, or tomatoes’. ‘Some people can’t eat goat meat, for example, my mother doesn’t eat meat at all, and when she eats meat she gets sick’. ‘My mother doesn’t eat meat either, when she eats beef she gets sick’. ‘We don't consume cotton oil, because it has burning effects’. ‘[We] do not eat eggs because they had hepatitis so they are not recommended to eat eggs as part of [our] diet’.	People that have illness of hepatitis and typhoid, they don’t eat *plov* because it’s heavy, they don’t eat eggs because they have diet’. ‘Some people if they eat lamb or eggs, it makes them sick. People with high blood pressure or heart problems. Goat meat gives us diarrhea’. ‘When you drink milk, you may get a headache’. ‘. Also not boiling water causes illness’.	‘*Osh* may cause pain in the stomach. When they eat grapes, it may cause bloating. If you eat pumpkin, it may cause burning in the throat’. ‘Sheep’s meat. It has many oils’.	There is little consistency between the three groups with respect to which foods can cause illness. However, in-married women, MiLs, and husbands all mention oily food as a potential source of illness. The greatest similarity in responses is observed between in-married women and MiLs with respect to spoiled foods, contaminated water, heavy/ oily foods, or increased sensitivity to some foods if an individual has specific health problems. In-married women, in particular, emphasize allergies as a cause of illness. Health problems that increase likelihood of illness that were mentioned by both in-married women and MiLs include: high blood pressure and hepatitis. In-married women and MiLs also noted hepatitis patients and people with high blood pressure have special diets. In-married women reported the greatest diversity of illness-causing foods; mentioning heavy, high-oil foods, eggs, and meat. Interestingly, in-married women also mention foods with chemicals/grown using fertilizers as sources of illness.
Health beliefs	‘We don’t eat imported chicken’. ‘We give children under one cow’s milk. Not goat’s milk. It’s nice; goat’s milk is nicer than cow’s milk’. ‘My baby was four months old, and I gave him cow’s milk because I didn’t have enough milk’. ‘The foods with vitamins, they eat them’. ‘The fruits they have vitamins. Cabbage, flour, I dry eggplants in summer, and then I eat it [them] in the winter’. ‘Also important are meat and eggs because they are healthy’. ‘Oranges, we don’t eat it makes the pressure down. Also the canning that is very cold, we don’t eat them in the winter’. ‘We do compote, we do canning with tomato and cucumber If we have money in the budget we buy meat’. ‘A light meal is soup, rice soup, noodles, rice porridge for breakfast’. ‘It’s important to have them clean, if we wash them properly that’s good for health’. ‘Using fertilizer less, using compost instead of chemicals’. We don’t eat imported chicken; we eat our chicken from our houses but we don’t eat imported chicken’. ‘If you have a problem with the stomach you shouldn’t eat hard meals (like *osh*)’. ‘My mother cannot eat *plov* because she has an allergy for rice’. ‘I am not in condition to buy formula, but I buy cow milk for my children which may be healthier’.	‘Onion, potato, flour, rice, carrot, beet, radish, sugar, oil, spaghetti, sugar, and salt are all important’. ‘Soup and *plov*, with rice radish, turnip, carrot, salads form carrot, we make salad from green radish, beetroot is good’. ‘They make salad from beet root as well’. ‘Sometimes we have problems buying meat’.	‘Some of them have high blood pressure, so if they have it they may not eat sugar. It depends on the disease’. ‘There are meats we do not eat for religious reasons’.	Affordability is mentioned as a barrier to healthy foods. Meat in particular may not be purchased due to affordability, though it is recognized by both MiLs and in-married women as healthy. Husbands, in contrast, state that meat may not be consumed for religious reasons. In-married women specify which foods are high in vitamins (fruits and vegetables). Both in-married women and MiLs cite the same basic foods that are bought at market (sugar, oil, flour, rice, salt…). In-married women in particular mention avoiding imported chicken for consumption. In-married women also detail the use of soft foods and cow’s milk for complementary feeding practices of infants. One in-married woman cited the use of cow’s milk in lieu of formula due to affordability. Both husbands and in-married women note the need for a modified diet if one has high blood pressure. Out of all of the groups, in-married women most strongly emphasize the consumption of vegetables, fruits, and dairy. The main constant is the mention of vitamins and vegetables and affordability challenges regarding meat between both groups of women.
General diet	‘Flour is the most important food. [As well as] oil, potato, onion, salt, salt, tomato pasta, beans’. ‘Vegetables are also important, cilantro is important, salad is important, cucumber, tomato, carrots, carrot salad, turnip, pepper, eggplant, pumpkin, rice’. ‘Pumpkins are healthier than meat’. ‘Meat is more nutritious than beans, turnip, herbs, pumpkin; many people have a diet from beans’. ‘Dill and cilantro’. ‘Salt is also important; tea is important’. ‘Banana, kiwi, pineapple, for the holidays’. ‘Almonds, walnuts, red beans they are very nutritious’. ‘In the past we didn’t consume sour cream, 10–15 years ago we didn’t make salads, and now in the past years we make salads with beetroot’. ‘We didn’t have pineapple, kiwi, oranges, banana’. ‘We consume eggs in a different way, we boil it, we fry eggplants with eggs and we cook some other different kinds of food with eggs’. ‘We didn’t used to eat bananas. I tasted [it] this year; I think that our fruits are tastier than the imported fruits. Our apples are tastier because they are ripened in the sun’. ‘I learned to can 2 years ago, there was one organization that came and demonstrated how to do it’.	‘Oil, onion, tomatoes, carrots, beans, rice, lentil, kidney bean, mung bean. Meat, sometimes’. ‘Grape carrot, persimmon, pomegranate, oranges, bananas. Sometimes we get banana’. ‘Rice porridge is good for people who have heart problems’. ‘In winter, rice porridge. It is called *shala’*. ‘Osh is also good because it makes people strong. Also, different types of fruits have different vitamins’. ‘We buy apple, orange, mandarin, date/fig because they are healthy and have vitamins. They are very healthy for pregnant women’. ‘Last year, I tried kiwi and banana for the first time. Kiwi for me as well last year. Also, pineapple’.	‘Flour, oil, tomato, onion, leafy vegetables, herbs. Tea. Flour and oil are most important. We eat lots of bread’. ‘Milk products. Traditional foods like *osh*. For breakfast [we] prefer milk products. Lunch [we] prefer soup. Dinner [we] like traditional food like sauce or stew’. ‘Potato, onion, flour. Oil, sugar, fruits for children. Pasta is also extremely important to us. They are important for preparing daily meals’. ‘Milk products. Fruit- apple, cereals. Meat, specifically cow meat’.	Across all three groups, flour, and oil are identified as the two most important staple foods. Next, onions, tomatoes, potato, and ‘fruits and vegetables’ (in general) are mentioned by all groups. In-married women emphasize the importance of beets, carrots, radish, salt, and sugar. Both groups of women detail various types of fruits and vegetables and methods of preparation. In-married women, in particular, discuss preparation of salad using beets. Furthermore, banana, kiwi, and pineapple appear to be recent additions to the Tajik diet according to two women's focus groups. Root vegetables appear to play particular importance according to women, as well as legumes and beans. In-married women and husbands mention the use of herbs, in-married women giving detail about preference, while MiLs do not. Only husbands cite dairy products as part of the general diet. Furthermore, husbands mention ‘traditional foods’ as important to the diet.

### Theme 1. Decision-making around food

All participant groups discussed consistent decision-making processes surrounding food, revealing clear gender roles in food preparation and purchase. Three subthemes were identified within Theme 1 including 1) purchasing foods, 2) cooking, and 3) food purchase decisions.

#### Purchasing foods

All three participant groups agreed that men are the primary purchasers of food. Women play a secondary role in food purchasing; women’s purchases are dependent on whether the husband is present. If men are unavailable to go to market, women will go to market and purchase food for the family. Women in the household (in-married women and MiLs) stated that men may be unavailable to purchase foods ‘if [they] are busy’. In other cases, women explained that seasonal migration of men to Russia has resulted in the increased role of women in food purchasing.

#### Cooking

There was a consensus across the study population that women, either the in-married woman or MiL, prepare food for the family. Furthermore, all three participant groups emphasized the role of the in-married women in food preparation. In this way, younger women fill the majority of cooking needs for the family while MiL plays a supportive role. For example, one MiL reported that when her daughter-in-law visits her family, the MiL takes over responsibility for cooking during that time. According to the combined reports of the MiLs and husbands, men may depend on the MiLs to cook, may eat out, or cook food themselves if the wife is not available to cook.

#### Food purchase decisions

Again, all participant groups agree that women, either the in-married woman or MiL, decide which foods to buy. According to the participants in the MiL sessions, the reason women decide which foods to purchase is that their ‘husbands don’t know what to buy because [they] cook’. In cases where MiL and in-married women cohabitate, both women play a role in the decision-making process. However, if MiL and in-married woman live separately, each woman decides what foods to purchase for her respective household.

### Theme 2. Accessibility of food

In general, in-married women, MiLs, and husbands showed consistency in their responses regarding accessibility of food. There are some key differences, however, with respect to where food is acquired, when food is available, how it can be obtained, and solutions for navigating challenges regarding food access. Furthermore, strategies for dealing with limited income were mentioned in three out of the four subthemes. Theme 2 has been divided into the following four subthemes: 1) food acquisition, 2) seasonality, 3) obtaining food, and 4) barriers and solutions.

#### Food acquisition

This subtheme is especially relevant because it illustrates where households are getting their food from. In general, the participant groups agreed that the majority of food is purchased at the market. However, MiLs also discussed occasional purchase from neighbors. In-married women emphasize the importance of homestead production for the majority of food needs, utilizing market purchases only for food items not produced on their own land. Cooking basics, including flour, oil, and onions, were among the food items that must be purchased.

#### Seasonality

There was unanimous agreement among in-married women, MiLs, and men that winter is the most challenging month with respect to food access. All participant groups discussed a strong dependence on food purchases during the winter due to the seasonality of home gardens. According to husbands, this was sometimes due to insufficient Fall harvests. Spring was recognized as the second-most difficult season, with some need to purchase foods. Summer and, to a lesser degree, Fall were identified as seasons of surplus in which nearly all food is produced in home gardens, ‘so there is no need to go to the market’.

#### Obtaining food

This subtheme indicates how households obtain their food, whether it is through trade, money, or potentially other means. Women (in-married women and MiLs) agreed that food is primarily obtained through monetary purchase. However, in-married women emphasized the importance of informal credit agreements in which they purchase food on account (without transfer of money) and pay the supplier later. This was mentioned as being quite common in village markets; however, in district markets (which were larger and more centralized within a region) this was not acceptable. While food trade is recognized as rare by both in-married women and MiLs, husbands identified trade as the ‘typical’ means of obtaining food and ‘other times [they] pay with money’.These responses reveal gender-based differences in market transactions in which husbands are more likely to trade than women.

#### Barriers and solutions

All three participant groups identified lack of money as a barrier to food security. In-married women and MiLs reported limited employment as drivers of low income. MiLs in particular suggested skill-building workshops for young girls as a potential means of increasing household income. In-married women and husbands additionally focused on limited food due to poor-quality harvests and low autumn yields. In-married women discussed pest management as a potential solution to improving the quality of crops and reducing insect-related damage. Husbands and MiLs emphasized canning and pickling as solutions for minimizing food purchases and maintaining food sales through value-added goods during the winter.

### Theme 3. Misconceptions around food

In general, the participant responses regarding misconceptions around food were consistent regarding which foods to avoid. However, the responses were inconsistent regarding the *reasons* for avoiding that food. Inconsistencies were more apparent in the comparison of women and men focus groups; MiLs and in-married women showed greater congruency than husbands. Theme 3 includes two subthemes: Pregnant women and children under five.

#### Pregnant women

All participant groups discussed restrictions in carbohydrate intake as an important strategy for prenatal health. However, these beliefs vary in the way they were presented. For example, in-married women and MiLs emphasized restrictions in carbohydrate consumption by mentioning a wide range of foods that pregnant women should avoid including *mantou*, noodles, bread, baked goods, and *osh*.^2^ Husbands illustrated the practice of prenatal limitation of carbohydrates by identifying *osh* as a food that pregnant women avoid. MiLs and in-married women gave a highly consistent reason for avoiding carbohydrates during pregnancy: carbohydrate consumption leads to a heavy birth weight and difficult delivery. Furthermore, in-married women and MiLs identified doctors as the source of this information.

Aside from carbohydrate consumption, both women participant groups agreed that women should monitor their intake of ‘heavy’ foods including fats, oils, and nuts because these foods can increase morning sickness symptoms. In-married women did not mention meat consumption, while MiLs and husbands recommended meat and eggs for a prenatal diet. Husbands also discussed avoidance of particular meats (rabbit and horse) due to the increased risk of cleft pallet in infants (See Table 1, ‘Husbands’), whereas only MiLs discussed the importance of dairy consumption during pregnancy.Interestingly, both women participant groups discuss ideas surrounding ‘pure food’. In-married women, for example, referred to avoiding food that was produced using pesticides or fertilizers while MiLs discussed consuming domestic eggs ‘but not the imported ones’. MiLs further identified food from China as unhealthy.

#### Children under five

While there are diverse opinions regarding proper young child-feeding practices between in-married women, MiLs, and husbands, all three participant groups agree that children should not receive eggs. However, each group cited a different reason for the practice. For example, MiLs stated that eggs are ‘hard for the stomach to digest’, while husbands reasoned that eggs contain too many calories. One in-married woman, meanwhile, highlighted her child’s taste preference as the primary reason for not feeding her children eggs (See Table 1). Finally, in-married women and MiLs discussed contaminated water and unwashed fruits and vegetables as sources of diarrhea.

In-married women consistently mentioned two ideas concerning children’s diets (1) children’s diets should include mostly light, soft foods and (2) the ability to consume a diverse diet depends on the child’s baseline health, or whether the child is perceived as ‘healthy’ or ‘unhealthy’ according to the community standard. With respect to the first idea, in-married women show a preference for feeding their children ‘light meals’ like ‘noodles and soup’ because they are perceived to be easier to digest than ‘heavy meals’, like fatty or oily foods. In addition, in-married women emphasize the need for children’s meals to be soft in consistency and texture. In-married women discussed preparation practices for soft foods, such as mashing potatoes, soaking biscuits in water, or pureeing apples. According to in-married women, baseline health affects child consumption patterns because healthy children are able to eat a greater diversity of food items with a reduced risk of becoming ill. Unhealthy children, meanwhile, must eat a more restricted (‘soft’ or ‘light’) diet to prevent further illness. In-married women explained that hard and heavy foods are difficult to digest and likely to make unhealthy children sick. In-married women also connected rural living and exercise to the general health of children (See Table 1, *children under five*).

### Theme 4. Dietary information

Participants have varying opinions surrounding dietary information, especially with respect to health beliefs and foods that cause illness. Theme 4 has been divided into three subthemes, including foods that cause illness, health beliefs, and general diet.

#### Foods that cause illness

Responses within this subtheme are highly diverse. However, there was a consensus across all three participant groups that oily foods are a source of food-related illness. The greatest similarity in responses is observed between the women participant groups (in-married women and MiLs). For example, both women’s groups agreed that spoiled foods, contaminated water, and heavy foods cause illness. Furthermore, both groups noted that individuals with pre-existing health problems^3^ and elderly people experience an increased risk of food-related illness and require dietary restrictions to avoid frequent health issues. In-married women listed the greatest diversity of illness-causing foods. Finally, in-married women emphasized the impact of allergies on illness and diet.

#### Health beliefs

Participant groups showed differing opinions with respect to health beliefs. However, all groups mentioned the importance of traditional foods, many of which are high in carbohydrates and utilize either rice or flour as their main ingredient.^4^ In general, in-married women and MiLs show the greatest consistency in their responses, citing the importance of vegetables and fruits due to vitamin content. In addition, both MiLs and in-married women discussed affordability as a barrier to purchasing meat, while husbands specifically discuss religion as a barrier to the consumption of certain meats. Husbands and in-married women identified the need for individuals with high blood pressure to have modified diets through lower sugar and carbohydrate consumption.

In this subtheme, in-married women again mentioned avoiding foods produced using fertilizer or chemicals and their preference for domestic chicken and meat. This concept was first discussed by in-married women (fertilizers) and MiL (domestic animal sourced foods) in Theme 3 (Misconceptions around food, *pregnant women*). Interestingly, in-married women emphasize the importance of ‘clean’ food, stating that ‘if we wash [the vegetables] properly, it is good for our health’. In-married women and MiLs first discussed the importance of washing fruits and vegetables within Theme 3, *children under five.*

#### General diet

Across all three participant groups, flour and oil are identified as the most important staple foods. In addition, onions, tomatoes, potato, and ‘fruits and vegetables’ in general were reported by all groups. Both women participant groups detailed various fruits and vegetables along with methods for preparing them. Furthermore, both women’s groups participants identified banana, kiwi, and pineapple as recent introductions to the Tajik diet. Lastly, both groups discussed the important role of root vegetables, legumes, and beans in the general diet. In-married women and husbands mention the use of herbs, and in-married women highlighted taste preference as a driver of food choice. Interestingly, only husbands cited dairy products as an integral part of the general diet.

## Discussion

As prior research establishes, malnutrition is a critical public health issue that disrupts the livelihoods of many Tajik people (2). Stunting, iodine deficiency, and maternal and child anemia account for the greatest proportion of negative health outcomes due to undernutrition in Tajikistan (11). Poor diet, characterized by insufficient amounts of nutritious foods consumed at low frequency, has been identified as the primary driver of such conditions (15). Undernutrition is concentrated among women of reproductive age, children, and rural populations, which may lead to immediate and long-term productive and reproductive consequences and hold severe implications for the quality of life in rural areas. The Tajik diet includes inhibitors of nutrient absorption (particularly iron) like black tea and exclusion of absorption enhancers, such as fresh fruit, animal source foods, and vegetables, which are seasonally unavailable or unaffordable according to this study (15, 16). Even so, these findings point to a general knowledge about the health benefits of various fruits and vegetables, especially among women. Participants cited onion, tomato, and potato as essential components of their diet, and placed importance on the health benefits provided by beans and many types of fruits and vegetables. Many women participants (both in-married and MiLs) placed additional emphasis on consuming a healthy diet during pregnancy. Many fruits and vegetables are considered indulgences rather than central components of the Tajik diet, unlike everyday staples. Participants pointed to the high cost associated with imported fruit, such as kiwi, as a major barrier for why they do not include more fruits and vegetables in their diet. As a result, many fruits are exclusively reserved for special occasions – as one participant stated, residents in her village saved ‘banana, kiwi, and pineapple for the holidays’. It is customary in Tajikistan to bring food to guests, but there is concern that this expectation for the appearance of wealth leaves less food on the family table, as mentioned in one of the husband focus groups. To encourage greater dietary diversity, it is recommended to promote locally sourced, affordable health options to make healthy choices more commonplace.

Clear gender roles were noted for the following subthemes of Theme 3, Decision-making around food: *Purchasing food*, *cooking*, and *food purchase decisions*. Husbands appear to hold the responsibility of going to market to purchase or trade for food, while women (MiLs and in-married women) make the decisions regarding what type of food to buy. According to women, the responsibility of making food purchase decisions resides with women because they are the primary food preparers. Between the women of the household, it is unclear under what conditions food purchase decisions are allocated to the MiL as opposed to the in-married woman. The responsibility of making food decisions appears variable, in some cases dependent on the availability of the in-married woman and in others based on negotiations between the in-married woman and the MiL. The MiL may also have a transient role in food preparation depending on the availability of the in-married woman. FGDs also indicated that the absence of men within communities as a result of migration impacted daily life and household roles significantly. Although the focus group instrument did not specifically ask questions about male migration, female FGD participants brought up migration several times as it related to other questions surrounding household decision-making. When asked about household responsibilities, such as shopping for food, women as a whole routinely mentioned how the absence of men had shifted the responsibility to them. MiL participation in shopping for food is significant with regard to the household’s nutrition status, as it seems to provide a greater opportunity for women to make changes in their diets. Since women are often the ones purchasing (MiL) and cooking (in-married women) food using gender-specific interventions that address diet diversity, specifically by introducing new crops, could prove more successful. Nevertheless, an increase in responsibilities as a result of male migration may not directly impact power within the household.

Based on the FDGs, it appears that migration has affected women’s lives to varying degrees, which depends significantly on the circumstances they are in. In addition, in cases where in-married women live with their MiLs, the MiLs were the heads of household when men were not present. Consequently, extension services should attempt to incorporate MiLs into trainings and educational interventions, as they are often the ones with a significant amount of power and time within a household. It is also beneficial to recognize that the amount of agency a mother has within her household will vary based on which family members she lives with.

Perhaps, the most striking findings from this study are the dietary practices and misconceptions about food prescribed to young children and pregnant women. As with previous studies conducted in other geographic regions, food taboos tend to have the greatest impact on the nutrition status of women and children (17). There was a general consensus that pregnant women should avoid carbohydrates and oily foods due to the risk of a difficult labor associated with heavier birthweight and irritation of morning sickness symptoms, respectively. For example, both in-married and MiLs reported that pregnant women should not consume carbohydrate-rich staple foods like *osh* and *mantou*. Husbands echoed these beliefs to a lesser degree, and with mixed opinions. For example, one participant claimed that ‘pregnant women eat everything’, while another agreed with the beliefs of women’s groups stating that ‘pregnant women do not eat *osh*’. Still other men reported a wide variety of beliefs incongruent with other participant groups. Women (MiLs and in-married women) appear to be the primary endorsers and implementers of dietary practices that limit carbohydrate consumption as indicated by the high degree of consistency in the food practices reported by women participant groups.

As many staple foods are rich in carbohydrates, women report the exclusion of these items during pregnancy. Exclusion of staple foods may hold significant implications during lean seasons (Winter and Spring) when vegetables and fruits are unavailable and households live on reduced incomes. The FGDs did not reveal whether such limitations on pregnant women’s diets are altered during the lean season to accommodate reduced access to alternative foods. While beliefs surrounding carbohydrate intake and heavy birthweight have not been previously investigated in Tajikistan, studies from other geographic regions (Ethiopia and Nigeria) have made similar findings (16, 18, 19). In Nigeria, one study revealed that pregnant women who were not practicing taboos had significantly greater weight gain and gave birth to heavier infants as compared to women practicing food taboos (17). Determining the immediate impact of food taboos on pregnant women in Tajikistan may represent opportunities for improving maternal health outcomes including maternal morbidity and preterm birth complications.

Restriction of oily foods – oily food in the FGDs included certain meats (goat, lamb) as well as foods containing or cooked with vegetable oil – has additional implications on pregnancy outcomes and child development. For example, sources of omega-3 fatty acids (fish, nuts, vegetable oil) and animal source food (meat) are shown to benefit fetal (physical and cognitive) growth and support the increased nutrient demand associated with pregnancy (20). Avoidance of oil during pregnancy appears to be a cultural practice, while exclusion of carbohydrates was learned through counseling with doctors. This may indicate miscommunication or misunderstanding of dietary recommendations between patients and healthcare professionals.

FGDs conducted with women revealed consistent beliefs surrounding child feedings practices. For example, both MiLs and in-married women voiced that children should receive ‘light’ and ‘soft’ foods – described as foods containing less fat and carbohydrates, and foods that have been watered down to a soft consistency. Among the foods restricted from children’s diets are staple foods like *sambusa*, *osh*, *fatir*, and *mantou*, which are described as ‘hard meals’. According to women participants, heavy foods are more ‘difficult to digest’ and may result in stomach problems. Interestingly, these beliefs were not reflected by FGDs conducted with husbands. Finally, in-married women and, to a lesser degree, MiLs were concerned with diarrheal disease in children. Both reference cucumber and water as sources of diarrhea; In-married women additionally reported that unwashed fruits and vegetables may be a source of diarrhea. Additional research is needed to determine whether the practice of restricting staple foods among children contributes to the prevalence of stunting in Tajikistan.

It was mentioned in several FGDs with in-married women that that they gave infants aged under 6 months cow’s milk when they had difficulties with breastfeeding. Various reasons for the use of cow’s milk included not having enough breast milk or not having enough money to buy formula. There was a belief that cow’s milk was comparable to breast milk and made babies strong. When asked if participants used milk from other sources, such as goats, in-married women indicated that only cow’s milk was preferred because of availability and the high cost of formula. Due to the dangers of using unmodified cow’s milk for infants aged under 9 months, nutrition-specific interventions should be introduced to address the use of alternative breast milk within the rural regions. In order to reduce breastfeeding cessation, potential interventions could include providing lactation and breastfeeding support.

Due to the small sample size of this study, several limitations need to be considered. Consistency of beliefs around access to foods or food misconceptions may vary according to the participant’s proximity to food centers or education status, respectively. Moreover, the geographic distribution of selected villages was shaped around prearranged Women’s Economic Empowerment Project (WEEP) group meeting times and consisted mainly of participants from these groups. Also, while the sample population was intended to include more male focus groups, due to the lack of young, adult males in the target areas, researchers were unable to have a more balanced representation.

This study demonstrates the need for gender-responsive solutions using agricultural extension agents to counter barriers to a healthy diet and dietary diversity in Khatlon Province. Results also established a link between emigration of males to Russia for work and changing household social structure. The impact of migration on household decision-making is worth exploring further to offer more mindful approaches for future intervention strategies in the province. This study suggests that women hold the majority of nutritional knowledge and misconceptions surrounding food, both practicing and engaging in food-restrictive taboos alongside a genuine knowledge of healthy eating practices. Overall, men lacked knowledge of nutrition and dietary practices. In order for agriculture extension agents to address nutritional status of their beneficiaries as part of their duties, extension agents must have knowledge of the intra-household decision-making processes between women and men and between MiLs and in-married women that determine what foods are available in the household. In addition, a food recall study may be a comprehensive follow-up to glean a more accurate picture of the dietary patterns in the Khatlon Province. Further investigation may also prove useful in assessing if there exists an effect of village proximity to district markets on food access.

## Footnotes

1 The two focus groups that were excluded from the analysis were not stratified, including both in-married women and MiL, and had more participants than necessary (14 in one and 18 in the other).

2 Mantou (dumpling) and osh (pilaf) are traditional Tajik foods prepared with flour and rice, respectively.

3 Heart problems, high blood pressure, allergies, hepatitis, and typhoid were among the health problems mentioned.

4 Husbands identified these foods by simply stating ‘traditional foods’ while in-married women and MiLs discussed their preparation in detail. In-married women and MiLs specifically mention preparation of osh, mantou, and shala.
